# Black Stain and Dental Caries: A Review of the Literature

**DOI:** 10.1155/2015/469392

**Published:** 2015-02-24

**Authors:** Tomasz Żyła, Beata Kawala, Joanna Antoszewska-Smith, Maciej Kawala

**Affiliations:** ^1^Department of Maxillofacial Orthopaedics and Orthodontics, Wroclaw Medical University, Krakowska 26, 50425 Wroclaw, Poland; ^2^Department of Prosthetic Dentistry, Wroclaw Medical University, Krakowska 26, 50425 Wroclaw, Poland

## Abstract

Black stain is characterized as a dark line or an incomplete coalescence of dark dots localized on the cervical third of the tooth. Over the last century, the etiology of black stain has been the subject of much debate. Most of the studies concerning this issue were conducted in pediatric population. According to the reviewed articles published between 2001 and 2014, the prevalence of black stain varies from 2.4% to 18% with equal sex distribution. The majority of the authors confirm the correlation between the presence of black stain and lower caries experience. The microflora of this deposit is dominated by *Actinomyces* spp. and has lower cariogenic potential than nondiscolored dental plaque. Iron/copper and sulfur complexes are thought to be responsible for the dark color. In patients with black stain saliva has higher calcium concentrations and higher buffering capacity. Factors such as dietary habits, socioeconomic status, and iron supplementation may be contributing to the formation of black stain.

## 1. Introduction

The causes of tooth discoloration are classified according to the location of the stain and are divided into extrinsic, intrinsic, or internalized. Extrinsic discoloration is deposited on the tooth surface or in the acquired pellicle. The compounds that are incorporated into the pellicle produce a stain due to either their basic color or chemical interaction at the tooth surface. Intrinsic stains occur when the tooth structure is penetrated by pigmented materials, usually during tooth development. Internalized discoloration is the incorporation of extrinsic stain within the tooth substance following dental development [1, 2].

A specific type of external discoloration is called black stain (BS). It is characterized as a dark line or an incomplete coalescence of dark dots formed on the cervical third of the tooth and following the contour of the gingival margin, firmly attached to the tooth surface. BS is a common finding in children; however it can be also seen in adults [3]. Studies have shown equal prevalence in both sexes [4, 5]. The characterization of factors contributing to the formation of BS and its nature has become of interest since association between the presence of BS and lower caries experience in children was noted.

BS is considered to be a special form of dental plaque with a tendency for calcification [3, 6]. The ultrastructural examination of this deposit revealed microorganisms embedded in matrix. Almost all of bacteria were Gram-positive rods [7]. The microbiological composition of the BS is thought to be dominated by* Actinomycetes* [8]. Recent PCR analysis of plaque samples of children with BS showed significantly higher number of* Actinomyces naeslundii* and lower number of* Lactobacillus *spp. than in nondiscolored plaque samples [9]. The pigment is suggested to be a black insoluble ferric compound, probably ferric sulfide formed by the interaction between hydrogen sulfide produced by bacteria and iron [3]. The studies on the composition of BS disclosed higher content of calcium and phosphate than in nondiscolored plaque. Spatial chemical analysis using wavelength dispersive spectrometry showed corresponding areas of high concentration of sulfur and copper/iron. This may suggest that sulfur and metal ions form intensely colored compounds [6, 10]. Little data is available on chemical composition of saliva in subjects with BS. However, higher levels of salivary buffering capacity, higher pH, and increased concentrations of calcium and phosphate were reported [11–13].

The prevalence of BS varies between 2.4% and 18% because of unspecified criteria used for diagnosis and different populations included in the studies. Most of the authors showed that the presence of BS is associated with lower caries experience. The causative factors of BS are not fully understood. Certain types of bacteria seem to be involved in the etiology. It is not clear how the presence of BS on the tooth surface reduces susceptibility to caries. The dominant occurrence on smooth surfaces was not associated with lower caries experience on these surfaces which implies that caries resistance in children with BS is a result of a general lower caries activity rather than a localized effect [4, 5, 9, 14–19].

## 2. Search Strategy

We searched MEDLINE using the terms “tooth discoloration,” “black stain,” and “dental plaque.” No language restriction was applied. We also searched the reference lists of included articles and selected those we judged relevant. A comprehensive literature research was performed up to May 2014.

## 3. Black Stain Prevalence and Caries Status 

The origin of BS and its caries-protective properties have been discussed for over a century. In 1890, Miller noted the presence of BS in members of the same family and suggested hereditary constitutional factor. At the beginning of the 20th century, Pickerill described the plaque as a thin dark brown stained line about the necks of the teeth, appearing in the form of a deposited film of calculus. His suggestion that the occurrence of BS is a sign of immunity to caries highlighted the need for further research in this area [20]. We summarized findings on the epidemiology of BS and its relationship with caries prevalence and experience from 2001 to 2014 (Table 1).

**Figure 1:**
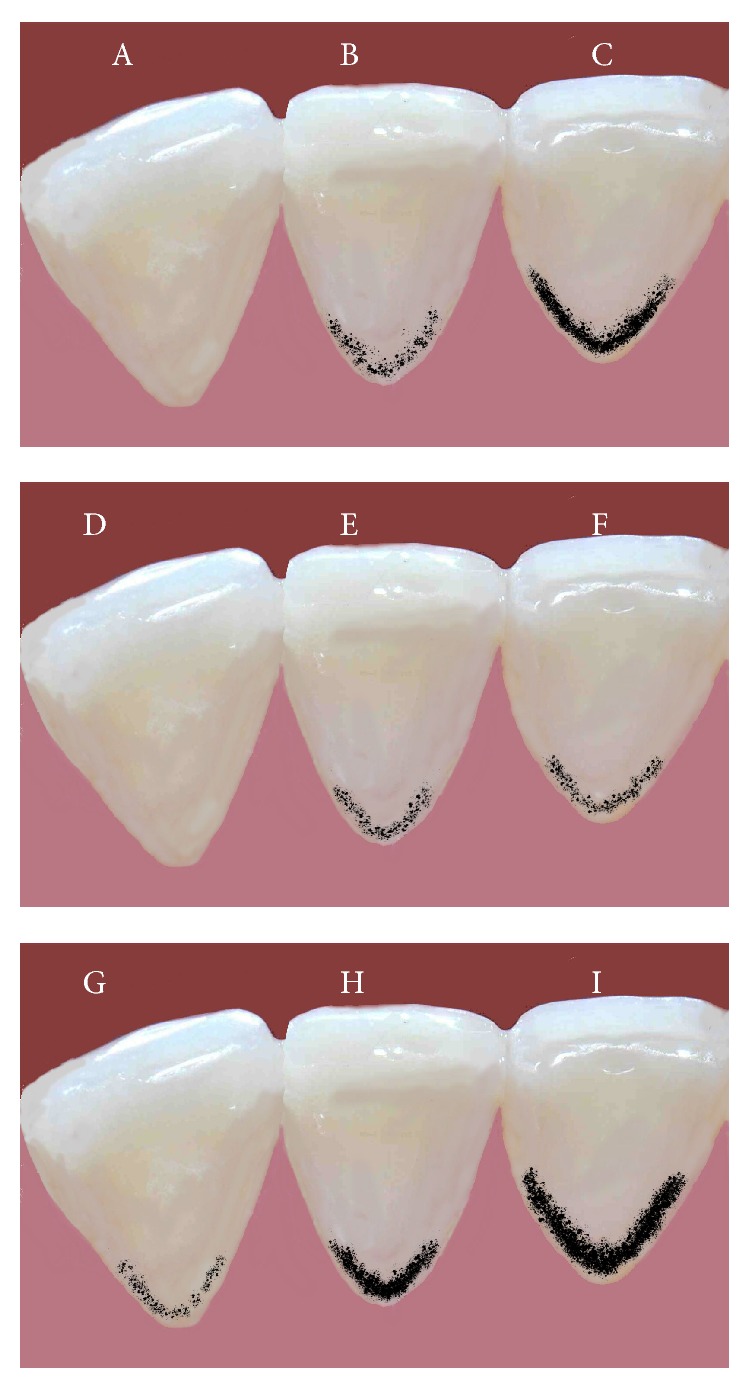
Black stain classifications. (A)–(C) Criteria according to Shourie, (D)–(F) criteria according to Koch et al., and (G)–(I) criteria according to Gasparetto et al.

Criteria for the diagnosis of BS are not well established. Shourie used the following criteria for classifying BS: (1) no line, (2) incomplete coalescence of pigmented spots, and (3) continuous line formed by pigmented spots [23]. Koch et al. introduced new diagnostic criteria. They described the presence of BS as dark dots (diameter less than 0.5 mm) forming linear discoloration (parallel to the gingival margin) at dental smooth surfaces of at least two different teeth without cavitation of the enamel surface [15]. Additional criterion based on the extension of the tooth surface area affected was created by Gasparetto et al. Score 1 corresponded to the presence of pigmented dots or thin lines with incomplete coalescence parallel to gingival margin; score 2 corresponded to continuous pigmented lines, which were easily observed and limited to half of the cervical third of the tooth surface; score 3 corresponded to the presence of pigmented stains extending beyond half of the cervical third of the tooth surface [18]. Described black stain classifications are presented in Figure 1.

Koch et al. examined 1086 children aged 6–12 years in Potenza, Italy. Four examiners were calibrated according to World Health Organization (WHO) criteria for caries diagnosis. BS diagnosis was established according to Koch et al. criteria. The prevalence of BS was 6.3%. The mean Decayed/Missing/Filled Teeth (DMFT) score was 0.49 ± 1.05 for children with BS and 0.97 ± 1.40 for children without BS (*p* < 0.007). Children with BS had fewer decayed permanent teeth than the other children (*p* < 0.001). No correlation was found between the presence of BS and age [15].

The study on 263 children aged 6–12 years was conducted in a small Brazilian city of Porto Rico (2600 inhabitants) by Gasparetto et al. The examinations were performed by four dentists, who had been previously trained and calibrated for WHO criteria for caries diagnosis. The diagnosis of BS was established according to criteria described by Shourie, Koch et al., and the authors of the study. Examinations were conducted in school setting and only permanent dentition was evaluated. The prevalence of BS was 14.8%. The number of children with caries-free permanent dentition was not statistically different between groups. The study did not show any statistical difference between caries prevalence and BS presence. According to the Gasparetto et al. criteria 41% of the children with BS were classified as score 3, 30.8% as score 2, and 28.2% as score 1. The mean DMFT was 1.46 ± 1.39 in children with BS and 2.42 ± 2.09 in children without BS. The presence of black extrinsic tooth stain was negatively correlated with the severity of caries (*r* = −0.16; *p* < 0.05). In addition, a significant negative correlation between the severity of BS (score) and DMFT (*r* = − 0.16; *p* < 0.01) was observed [18].

Heinrich-Weltzien et al. carried out a study on 1748 children (mean age 11.7 ± 1.1) in rural areas of Philippines. Out of 32 schools included, 19 had participated for 5 years in a comprehensive school-based preventive program with daily tooth brushing, application of fluoride varnish three times a year, manual restorative treatment of permanent teeth, and extraction of nonrestorable teeth. At the time of study 7 schools had participated for 2 years in basic preventive program with daily tooth brushing and emergency oral treatment on demand. The other 6 schools were control for the intervention program. A subgroup of four remote schools was created. DMFT score was recorded in 1121 children and Decayed/Missing/Filled Surfaces (DMFS) score in 627 children. Children were examined by calibrated dentists, outdoors on school benches under direct sunlight. BS was recorded present or absent in the dentition. The prevalence of BS was 16% and did not differ between three intervention groups; however, it was significantly higher in remote schools (*p* < 0.05). The caries prevalence and caries experience were significantly lower (*p* < 0.05) in children with BS compared to children without this deposit. The distribution of DMFS between surfaces did not differ between the groups [14].

Bhat conducted a study in schools of Udaipur, India. The sample consisted of 1472 children aged 6 to 12 years (mean age 9.4 ± 1.9). The clinical examinations were performed by two trained and calibrated dentists under natural light in the school setting. The diagnosis of BS was performed according to Shourie, Koch et al., and Gasparetto et al. criteria. Only the permanent dentition was evaluated. The overall prevalence of BS was 18%. Caries prevalence and experience were significantly lower in BS group (45.1%; DMFT 1.12 ± 1.41) than in children without BS (60.1%; DMFT 1.77 ± 1.87). There was no difference between DMFS values on occlusal and smooth surfaces in both groups; however DMFS index on proximal surfaces was lower in BS group (*p* < 0.05). Extension of BS was evaluated according to Gasparetto et al. criteria. Most children were classified as score 3 (42.4%), 33.4% as score 2, and 24.2% as score 1. Negative correlation between severity of BS and DMFT (*r* = − 0.36; *p* = 0.001) was observed [19].

An epidemiological study conducted on 12-year-old Cypriot children showed that subjects with BS had lower mean DMFT compared with subjects without BS; however, this difference was not statistically significant (0.49 ± 1.00 and 0.66 ± 1.18, resp.; *p* > 0.05) [21].

Evaluation of BS prevalence and its association with caries in a group of 1120 5-year-old children was carried out in Pelotas, Brazil. The examinations were performed at homes by trained dentists. The presence of BS was recorded according to Koch et al. criteria and dmfs index was assessed. BS was diagnosed in 3.5% children. Although no statistical difference was observed in bivariate analysis, caries-free children showed higher prevalence of BS (4.0%; 3.0–5.3) than children with dmfs > 0 (3.0%; 2.0–4.1) [17].

Martin et al. examined 3272 6-year-old children in Oviedo, Spain. The patients were examined by one dentist in a dental office. BS was recorded present or absent; dmft index was used to measure caries experience. The prevalence of BS was 3.1% and did not differ between sexes. There was no association between BS presence and lower dmft index [5].

A study on a group of 950 children aged 3–5.5 years was carried out in Thessaloniki, Greece. The examinations were performed in kindergartens by one calibrated examiner. No information is given about criteria used for BS diagnosis; dmfs was used for caries assessment. The prevalence of BS was 2.4%. The dmfs score in children with BS was significantly lower than in children without BS (0.38 ± 0.9, 1.19 ± 3.9, resp.) [16].

Chen et al. conducted a study in Shanghai, China. A trained dentist scored caries status using dmft and dmfs indices. BS was assessed according to Koch et al. criteria and was observed in 138 of 1397 (9.9%) children included in the analysis. The mean age of children with BS was 4.55 years. The prevalence of caries in subjects with BS was significantly lower than in group without BS (46.4% and 59.1% resp.; *p* < 0.01). The mean dmft and mean dmfs were both significantly lower in children with BS (1.91 and 4.22, resp.; *p* < 0.01) compared with children without BS (2.97 and 6.69, resp.; *p* < 0.01). The differences in the distribution of caries on tooth surfaces were not statistically significant between the two groups [4].

The association between caries experience and BS prevalence was also examined in an adult population. All of the 280 patients aged 18 to 29 years old were young soldiers. DMFT was scored by one trained examiner. Mean DMTF was lower in BS group than in control group (4.2 ± 3.9, 6.0 ± 4.8 resp.; *p* < 0.001). A significant difference was also found in D component of DMFT score (1.6 ± 2.5 in BS group and 2.4 ± 3.5 in control group; *p* < 0.05) [22].

## 4. Factors Contributing to the Formation of Black Stain

Unclear etiology of BS makes it difficult to distinguish factors associated with its formation. Few authors attempted to find correlations between sex, age, diet, oral hygiene, socioeconomic status, medications, and BS prevalence. In all reviewed papers there was no association between sex and BS prevalence [4, 5, 15, 17]. Chen et al. showed that the occurrence of BS increases with age; however the correlation is not statistically significant. The authors also found that the number of stained teeth increases with age (*p* < 0.007) [4]. In one report, more stained teeth were observed in permanent dentition compared to primary dentition [24]. Most of the studies are conducted with children and there is no data about the prevalence of BS in adult population. Dietary habits may also play a role in the etiology. Consumption of vegetables, fruits, dairy products, eggs, and soy sauce promotes BS development [4, 5]. Children who had never been fed with nursing bottle tend to have higher BS occurrence [4]. Drinking tap water instead of bottled mineral or natural well water also seems to be associated with higher prevalence of BS in Brazil [17]. There is conflicting data on the influence of oral hygiene. Garcia Martin et al. reported that using fluoride toothpaste and mouthrinse containing fluoride encourages stain formation [5]. However, in another study there was no correlation between BS prevalence and the type of toothpaste or frequency of brushing. Interestingly, in patients with BS the mean VPI (visible plaque index) was lower than in the control group. Results about the influence of socioeconomic status on stain formation are dissonant. Some authors show that parents' low educational level is associated with higher prevalence of BS, whereas others report contrary findings [4, 17]. Iron supplementation during pregnancy and in childhood may also promote BS development [5, 25].

## 5. Chemical Composition of Black Stain 

Biochemical studies on BS composition confirmed higher calcium content in BS patients. This difference was statistically significant when a plastic instrument was used for sample collection but not a metal one. This substantiates the impression that the use of a metal instrument may increase the calcium and metallic ions levels. Phosphate concentrations were also higher in BS group [6]. Further research focused on the identification of the compound responsible for the black color of the stain. Scraped samples were subjected to qualitative chemical analysis. The results suggested that the black compound is probably ferric sulfide, formed by the reaction between hydrogen sulfide produced by bacteria and iron in saliva or gingival fluid [3]. Parnas et al. hypothesized that metallic ions found in BS come from the samples collection process. Therefore, the authors used both metal (study group B) and graphite (study group A) curettes to obtain the material. The chemical composition was assessed by the use of energy dispersive spectrometry (EDS). Group A compared to control group (without BS) had higher calcium and phosphorus levels. No differences were found in the amounts of the carbon, oxygen, sodium, magnesium, silicon, sulfur, chloride, and potassium. No traces of metallic ions were found in group A, whereas iron, copper, titanium, aluminum, and zirconium were detected in samples scraped with a metal instrument. This may suggest that the use of metallic instruments influences the sample composition [26]. However, Tantbirojn et al. performed a study in which they used extracted teeth with naturally formed black deposit, so sample collection bias could not have been introduced. Authors found traces of iron and copper. The spatial chemistry analysis showed that areas with high iron and copper concentrations corresponded with areas of high concentration of sulfur. This finding is in accordance with Reid et al. studies [3], suggesting that possibly metallic ions and sulfur complex are responsible for the black color of stains [10].

## 6. Salivary Parameters

Saliva plays an important role in maintaining oral health and protecting from dental caries. Salivary parameters such as pH, buffering capacity, and calcium and phosphate ion concentrations are well-known caries-protective factors [11–13]. There is little data describing the saliva composition in subjects with BS. Surdacka obtained saliva using paraffin wax chewing stimulation. Significantly higher levels of calcium, inorganic phosphates, copper, sodium, and total protein were found in patients with BS compared to controls. Glucose levels were significantly lower in BS group. No differences were found in iron, zinc, and magnesium concentrations. Authors also showed that pH was higher in children with stains; however salivary flow rate did not differ between the groups [11, 12]. Aysun et al. also studied salivary parameters in children with BS. Only few results were in accordance with the previous paper. Calcium levels and saliva buffering capacity were significantly higher in BS group. There were no significant differences in phosphorus levels and salivary pH. Salivary flow rate was lower in children with BS compared to children without BS [13].

## 7. Microbiology of Black Stain

Theliade et al. demonstrated that BS is a deposit consisting of microorganisms embedded in an intermicrobial substance with a tendency to calcification. Therefore, it can be classified as a type of dental plaque, although it is composed of different types of bacteria. Most of the microorganisms are Gram-positive rods [7]. Further studies aimed to isolate and identify the predominant cultivable microorganisms of the BS. As suspected Gram-positive rods were the largest morphological group. The majority of these were facultatively anaerobic and anaerobic rods, typical of* Actinomyces israelii *and* Actinomyces naeslundii *[8]. A PCR study designed to determine the presence of periodontal bacteria in BS showed that* Porphyromonas gingivalis* and* Prevotella melaninogenica* were absent in black deposit; however,* Actinomyces *spp. and* Aggregatibacter actinomycetemcomitans* were more prevalent in BS patients than in controls. This may suggest that these bacteria are involved in the formation of BS [27]. Another PCR analysis investigated BS samples for* Prevotella nigrescens, Prevotella intermedia, Actinomyces *spp., and* Streptococcus mutans. *Four analyzed bacteria had similar prevalence in both BS patients and controls. These findings are in contrast with other studies, in which* Actinomyces *spp. were the most prevalent species [28]. The most recent PCR study confirmed that* Actinomyces naeslundii* is more prevalent in BS patients. On the contrary,* Lactobacillus *spp. and* Fusobacterium nucleatum* can be found in higher numbers in subjects without BS. The authors also demonstrated that* S. mutans* tends to be more prevalent in BS-free samples. Additionally, no significant differences were observed between the prevalence of* A. actinomycetemcomitans *in BS and BS-free patients [9].

## 8. Conclusions

BS is a type of extrinsic tooth discoloration usually forming a line near the gingival margin. The prevalence of BS ranges from 2.4% to 18%. The predominant types of bacteria isolated from this deposit are* Actinomyces *spp. Compared to nondiscolored dental plaque BS contains lower numbers of cariogenic bacteria. The compounds responsible for the dark color are iron/copper and sulfur complexes. Saliva in BS patients has higher calcium concentrations and higher buffering capacity. Beneficial salivary parameters and noncariogenic plaque may explain the association between lower caries experience and prevalence in subjects with BS. Further research is needed to fully understand the nature, etiology, and caries-protective properties of BS.

## Figures and Tables

**Table 1:** Summary of epidemiological studies of black stain prevalence and its association with caries experience.

Study	Group (*n*)	Age (years)	BS diagnostic criteria	BS prevalence (%)	DMFT		dmft		Association between black stain and lower caries experience (permanent dentition)	Association between black stain and lower caries experience (primary dentition)	Area	Country
					BS group	Non-BS group	BS group	Non-BS group				
Koch et al. (2001) [15]	1086	6–12	Koch	6.3	0.49 ± 1.05	0.97 ± 1.40	1.87 ± 2.47	2.39 ± 2.62	Yes (*P* < 0.007)	No (*P* ± 0.05)	Urban	Italy
Gasparetto et al. (2003) [18]	263	6–12	Shourie, Koch, and Gasparetto	14.8	1.46 ± 1.39	2.42 ± 2.09	—	—	Not evaluated	—	Rural	Brazil
Heinrich-Weltzien et al. (2009) [14]	1748	11.7 ± 1.1	Present/absent	16	1.50 ± 2.10	2.50 ± 2.50	—	—	Yes (*P* < 0.05)	—	Rural	Philippines
Bhat (2010) [19]	1472	9.3 ± 1.9	Shourie, Koch, and Gasparetto	18	1.12 ± 1.41	1.77 ± 1.87	—	—	Yes (*P* = 0.001)	—	Urban	India
Panagidis and Schulte (2012) [21]	951	11.6	Present/absent	6	0.49 ± 1.00	0.66 ± 1.18	—	—	No (*P* > 0.05)	—	Rural/urban	Cyprus
França-Pinto et al. (2012) [17]	1120	5	Koch	3.5	—	—	3.3 ± 6.7	4.1 ± 7.4	—	No (*P* N/A^c^)	Urban	Brazil
Martin et al. (2013) [5]	3272	6	Present/absent	3.1	—	—	0.35 ± 1.12	0.65 ± 1.85	—	No (*P* = 0.47)	Urban	Spain
Boka et al. (2013) [16]	804	3.6 ± 1.3	Present/absent	2.4	—	—	0.38 ± 0.90	1.19 ± 3.90	—	Yes (*P* < 0.001)	Urban	Greece
Chen et al. (2014) [4]	1397	4.55	Koch	9.9	—	—	1.91 ± 3.08	2.97 ± 3.91	—	Yes (*P* < 0.001)	Urban	China
Shmuly et al. (2014) [22]	110 (BS^a^) 170 (NBS^b^)	22.0 ± 2.4 (BS) 21.0 ± 2.7 (NBS)	Present/absent	—	4.2 ± 3.9 (BS)	6.0 ± 4.8 (NBS)	—	—	Yes (*P* < 0.001)	—	—	—

^a^Black stain group; ^b^non-black stain group; ^c^data not available.
